# YAP and TAZ are dispensable for physiological and malignant haematopoiesis

**DOI:** 10.1038/s41375-018-0111-3

**Published:** 2018-03-26

**Authors:** Elisa Donato, Francesca Biagioni, Andrea Bisso, Marieta Caganova, Bruno Amati, Stefano Campaner

**Affiliations:** 1 Center for Genomic Science of IIT@SEMM, Fondazione Istituto Italiano di Tecnologia (IIT), Via Adamello 16, 20139 Milan, Italy; 2 Department of Experimental Oncology, European Institute of Oncology (IEO), Via Adamello 16, 20139 Milan, Italy; 3 Present Address: Division of Stem Cells and Cancer, Deutsches Krebsforschungszentrum (DKFZ), and Heidelberg Institute for Stem Cell Technology and Experimental Medicine (HI-STEM gGmbH), Heidelberg, Germany; 4 Present Address: Max Delbrück Center for Molecular Medicine, Berlin, Germany

YAP and TAZ are two transcriptional co-activators regulated by the Hippo pathway kinases (MST1,2 and LATS1,2), cytoskeletal tension, and several other signaling pathways [1–3]. As such, YAP/TAZ are emerging as key regulators of cell growth and tissue homeostasis, both in lower organisms and in mammals [3]. In particular, YAP/TAZ are activated in somatic stem cells to support self-renewal and pluripotency and, when ectopically expressed, can reprogram terminally differentiated cells into a stem cell/progenitor like state [4, 5].

Although a systematic genetic analysis is still missing, there is evidence implicating YAP/TAZ in the regulation of the haematopoietic system, both in physiological and in pathological conditions.

In cord blood-derived human haematopoietic stem cells (HSCs), TEAD1, the obligatory partner of YAP/TAZ, regulates differentiation in early B cells [3]. TAZ, but not YAP1, has been implicated in the lineage choice during naive T cell differentiation, where it functions as a coactivator of RORγt favoring pro-inflammatory TH17 differentiation over the immunosuppressive Treg fate [6]. This may also account for the reported role of the MST kinases (*Yap/Taz* inhibitors) in limiting autoimmune responses [7, 8].

While in solid tumors, YAP/TAZ are emerging as either potent oncogenes or downstream targets of oncogenic pathways, their role in haematopoietic malignancies appears to be context dependent. In multiple myeloma (MM) and leukemias, YAP seems to exert a tumor suppressive function by regulating the Abl1-dependent DNA damage response, which leads to apoptosis in cancer cells. This explains why deletion or downregulation of YAP/TAZ are frequently observed in MM and leukemias [9]. On the other hand, TEADs have been proposed to reinforce transcriptional activation of oncogenic programs and enhancer reprogramming during B cells transformation [10] and upstream Hippo pathway components are lost in leukemias and lymphomas [11, 12], thus arguing for a pro-oncogenic role of YAP/TAZ in some hematological malignancies.

To analyze the role of Yap and Taz in the adult haematopoietic system, we crossed mice double homozygotes for conditional knockout alleles of both *Yap* and *Taz* (*Yap*^*flox/flox*^*/Taz*^*flox/flox*^ mice) with *Mx1-CRE* mice (Supplementary Figure 1). Conditional activation of the *Mx1-CRE* led to an efficient and stable deletion of both alleles, thus ruling out counter selection of *Yap/Taz* floxed cells upon prolonged haematopoiesis (Supplementary Figure 2a, b). Blood analysis revealed no difference in the number of circulating white blood cells (WBCs) over time (Fig. 1a). Similarly, FACS analyzes of WBCs lineages confirmed the lack of alterations both in circulating and in bone marrow cells (BMCs) (Supplementary Figure 2). Red blood cells (RBCs) were largely unaffected by *Yap/Taz* loss, with the exception of a mild, decrease in RBCs count and hemoglobin concentration detected starting from 6 months post deletion (Fig. 1b, c). This was not paralleled by a statistically significant decrease in circulating erythroid progenitors (Ter119+ cells, Supplementary Figure 2g), arguing against a general defect in RBCs differentiation.

**Fig. 1:**
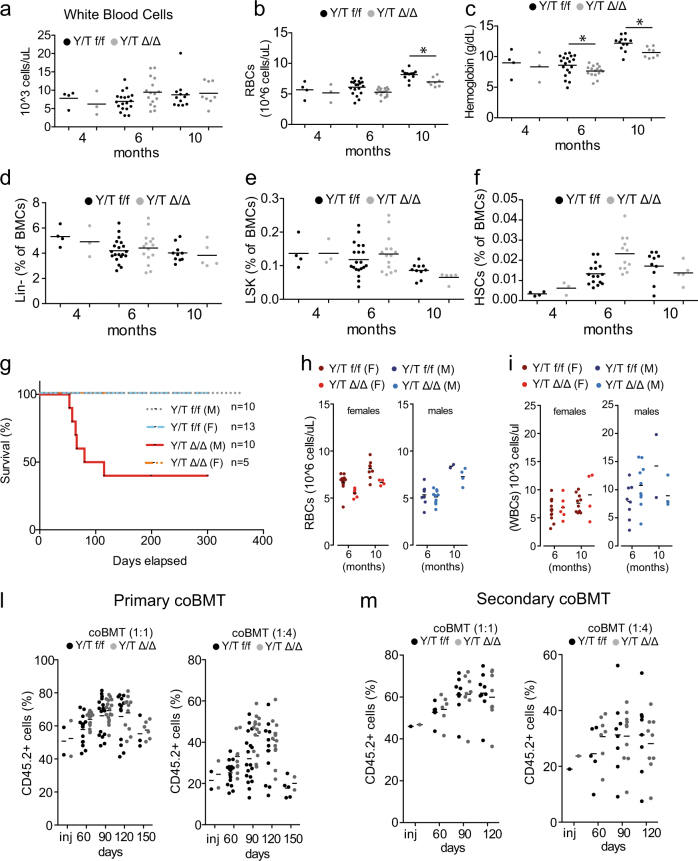
Analysis of peripheral adult haematopoiesis upon Yap/Taz loss. **a**–**c** Peripheral blood analysis of *Yap*^*flox/flox*^*/Taz*^*flox/flox*^ (Y/T f/f) or *Yap*^*Δ/Δ*^*/Taz*^*Δ/Δ*^ (Y/T Δ/Δ) mice. **a** WBCs, **b** RBCs, and **c** hemoglobin at 4, 6, and 10 months post recombination. **d**–**f** FACS analysis of BMCs: **d** Lineage-negative cells, **e** LSK progenitors, and **f** HSCs (Lin-, Kit+, Sca+, CD48−, CD150+, and CD34− cells). **g** Kaplan–Meier survival analysis (M: males, F: females). **h**, **i** Peripheral blood analysis: **h** RBCs and **i** WBCs. **l**, **m** coBMT experiments: **l** Chimerism assessed at different time points post-BMT. inj = donor cells mix. **m** Chimerism of the secondary coBMTs, as in **l**

FACS analyses revealed no difference in the frequency of early haematopoietic progenitor cells (Lin- and LSK) or in HSCs (Fig. 1d–f). Survival analysis showed a partially penetrant lethality of *Yap*^*Δ/Δ*^*/Taz*^*Δ/Δ*^ males (Fig. 1g). Importantly, these mice did not show any hematological defect (Fig. 2h, i and Supplementary Figure 3), or signs of progressive cachexia or anemia, which are typically associated with pan-cytopenia of the haematopoietic system. While the cause of lethality of *Yap*^*Δ/Δ*^*/Taz*^*Δ/Δ*^ males is still under investigation, it is most probably related to the activity of the *Mx1-CRE* in the liver.

**Fig. 2:**
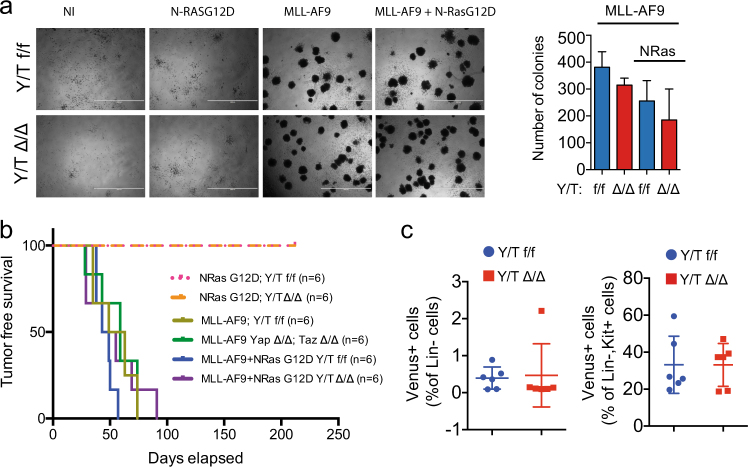
*Yap/Taz* do not contribute to MLL-AF9-driven leukemia. Modeling of MLL-AF9 and RAS-driven AMLs by viral transduction of haematopoietic progenitors from *Yap*^*flox/flox*^*/Taz*^*flox/flox*^ (Y/T f/f) or *Yap*^*Δ/Δ*^*/Taz*^*Δ/Δ*^ (Y/T Δ/Δ) mice. **a** Colony forming unit (CFU) assay (*n* = 6). **b** Kaplan–Meier analysis of mice xenografted (time = 0) as indicated. **c** Peripheral blood analysis of mice transplanted with HPCs transduced with MLL-AF9 cells (*n* = 6). Left: leukemic progenitors; right: leukemic stem cells

To test the role of *Yap* and *Taz* during enforced haematopoiesis, we performed competitive adoptive transfer experiments of BMCs (coBMT). Cells derived either from *Yap*^*Δ/Δ*^*/Taz*^*Δ/Δ*^ mice or from *Yap*^*flox/flox*^*/Taz*^*flox/flox*^ control (CD45.2+) were mixed with wild-type BMCs (CD45.1+) at different ratios (i.e., 1:1 and 1:4) and transplanted in lethally irradiated C57/Bl6 CD45.1 mice. Peripheral blood analysis showed that *Yap*^*Δ/Δ*^*/Taz*^*Δ/Δ*^ and *Yap*^*flox/flox*^*/Taz*^*flox/flox*^ BMCs reconstituted lethally irradiated mice equally well (Fig. 1l and Supplementary Figure 4).

Moreover, *Yap*^*Δ/Δ*^*/Taz*^*Δ/Δ*^ BMCs gave rise to differentiated cells of the myeloid and lymphoid lineages with the same efficiency of *Yap*^*flox/flox*^*/Taz*^*flox/flox*^ BMCs (Supplementary Figures 4 and 5). Coherently with these results, *Yap*^*Δ/Δ*^*/Taz*^*Δ/Δ*^ and *Yap*^*flox/flox*^*/Taz*^*flox/flox*^ BMCs contributed equally well to the stem cell and progenitors compartments, when transplanted in competition (Supplementary Figure 4c). Secondary coBMT experiments confirmed the haematopoietic proficiency of *Yap*^*Δ/Δ*^*/Taz*^*Δ/Δ*^ BMCs (Fig. 1m). Overall, these results suggest that *Yap/Taz* are dispensable for adult haematopoiesis and HSCs self-renewal. This is in line with similar studies conducted in other somatic tissues [3, 13] and reinforces the notion that YAP/TAZ might be more relevant in proliferative responses elicited by either regenerative or pathological cell growth. Obviously, our observations do not rule out a role for YAP/TAZ in lineage-restricted functions or their relevance in particular settings: an example is provided by the recent report showing how *Taz* regulates T-cell-mediated inflammatory responses [6]. Along the same line, *Yap* loss in myeloid cells potentiated the production of IFN-β and mediated immune responses to viral infections [14]. Thus, we believe our observations will be the ground for further investigation focused on more specialized haematopoietic functions.

To directly assess the role of YAP/TAZ in acute myeloid leukemia (AML) development, we took advantage of an established orthotopic mouse model of AML, based on the retroviral transduction of hematopoietic progenitor cells (HPCs) with the MLL-AF9 oncogene, alone or in combination with activated mutant NRAS (N-RAS^G12D^) (Supplementary Figure 6a). As expected, N-RAS^G12D^ transduction did not provide any growth advantage in *Yap*^*flox/flox*^*/Taz*^*flox/flox*^ cells; similarly no growth advantage was detected in *Yap*^*Δ/Δ*^*/Taz*^*Δ/Δ*^ HPCs transduced with N-RAS^G12D^ (Supplementary Figure 6b). MLL-AF9 conferred substantial growth advantage both alone or in combination with N-RAS^G12D^, with similar extent both in *Yap*^*flox/flox*^*/Taz*^*flox/flox*^ and in *Yap*^*Δ/Δ*^*/Taz*^*Δ/Δ*^ HPCs (Supplementary Figure 6b).

In line with these results, MLL-AF9 strongly stimulated the development of AML progenitors, with similar efficiency both in *Yap*^*flox/flox*^*/Taz*^*flox/flox*^ and in *Yap*^*Δ/Δ*^*/Taz*^*Δ/Δ*^ cells, while no CFU-stimulating activity was detected by N-RAS^G12D^ transduction in either control or *Yap/Taz-*deleted HPCs (Fig. 2a).

N-RAS^G12D^ HPCs did not give rise to AML once transplanted, regardless of their genotype, thus suggesting that *Yap/Taz* are not limiting factors (i.e., tumor suppressive) in RAS-driven transformation of HPCs (Fig. 2b). MLL-AF9 gave rise to AML with 100% penetrance and similar latency in both control and *Yap/Taz-*deleted HPCs (Fig. 2b). Accordingly, whole body leukemic cells dissemination was unaffected by *Yap/Taz* deletion (Supplementary Figure 6c, d) and leukemic progenitors and leukemic stem cells frequencies were similar in leukemias derived from either control or *Yap/Taz-*deleted cells (Fig. 2c). Overall, these results suggest that (i) *Yap/Taz* are dispensable for cell transformation and dissemination in MLL-AF9-driven leukemia and that (ii) *Yap/Taz* are not tumor suppressive in either N-RAS^G12D^ or MLL-AF9-driven AMLs. This suggests that the proposed gatekeeper function of YAP/TAZ [9] may be only relevant in selective settings, when specific genetic lesions may engage YAP/TAZ-regulated tumor suppressive mechanism(s).

Considering the growing interest in pharmacologically targeting YAP/TAZ to eradicate a variety of solid tumors, *Yap/Taz* dispensability in haematopoiesis has direct implications on the safety of such approach in terms of unwanted side effects, potentially detrimental for systemic haematopoiesis, which are frequently associated with cancer treatments. The hematological safety is further supported by the observation that deletion of *Yap/Taz*, despite their role as tumor suppressors in multiple myeloma and leukemias [9], does not predispose to spontaneous blood malignancies. The partial lethality of *Yap*^*Δ/Δ*^*/Taz*^*Δ/Δ*^ males, which is of non-hematological origin, is a cautionary note, which should be considered when *Yap/Taz* inhibitors will be evaluated in preclinical and clinical studies.

## Electronic supplementary material

supplemental figures

supplemental material and methods

supplemental table

## References

[1] Halder G, Dupont S, Piccolo S (2012). Transduction of mechanical and cytoskeletal cues by YAP and TAZ. Nat Rev Mol Cell Biol.

[2] Wada K, Itoga K, Okano T, Yonemura S, Sasaki H (2011). Hippo pathway regulation by cell morphology and stress fibers. Development.

[3] Yu FX, Zhao B, Guan KL (2015). Hippo pathway in organ size control, tissue homeostasis, and cancer. Cell.

[4] Panciera T, Azzolin L, Fujimura A, Di Biagio D, Frasson C, Bresolin S (2016). Induction of expandable tissue-specific stem/progenitor cells through transient expression of YAP/TAZ. Cell Stem Cell.

[5] Yimlamai D, Christodoulou C, Galli GG, Yanger K, Pepe-Mooney B, Gurung B (2014). Hippo pathway activity influences liver cell fate. Cell.

[6] Geng J, Yu S, Zhao H, Sun X, Li X, Wang P (2017). The transcriptional coactivator TAZ regulates reciprocal differentiation of TH17 cells and Treg cells. Nat Immunol.

[7] Du X, Shi H, Li J, Dong Y, Liang J, Ye J (2014). Mst1/Mst2 regulate development and function of regulatory T cells through modulation of Foxo1/Foxo3 stability in autoimmune disease. J Immunol.

[8] Salojin KV, Hamman BD, Chang WC, Jhaver KG, Al-Shami A, Crisostomo J (2014). Genetic deletion of Mst1 alters T cell function and protects against autoimmunity. PLoS ONE.

[9] Cottini F, Hideshima T, Xu C, Sattler M, Dori M, Agnelli L (2014). Rescue of Hippo coactivator YAP1 triggers DNA damage-induced apoptosis in hematological cancers. Nat Med.

[10] Hu Y, Zhang Z, Kashiwagi M, Yoshida T, Joshi I, Jena N (2016). Superenhancer reprogramming drives a B-cell-epithelial transition and high-risk leukemia. Genes Dev.

[11] Kim TS, Lee DH, Kim SK, Shin SY, Seo EJ, Lim DS (2012). Mammalian sterile 20-like kinase 1 suppresses lymphoma development by promoting faithful chromosome segregation. Cancer Res.

[12] Hartmann EM, Campo E, Wright G, Lenz G, Salaverria I, Jares P (2010). Pathway discovery in mantle cell lymphoma by integrated analysis of high-resolution gene expression and copy number profiling. Blood.

[13] Panciera T, Azzolin L, Cordenonsi M, Piccolo S (2017). Mechanobiology of YAP and TAZ in physiology and disease. Nat Rev Mol Cell Biol.

[14] Wang S, Xie F, Chu F, Zhang Z, Yang B, Dai T (2017). YAP antagonizes innate antiviral immunity and is targeted for lysosomal degradation through IKKvarepsilon-mediated phosphorylation. Nat Immunol.

