# Supplementary material for: YAP and TAZ are dispensable for physiological and malignant haematopoiesis
Source: Leukemia. 2018 Mar 26;32(9):2037–40. doi: 10.1038/s41375-018-0111-3 (PMC6127083; doi:10.1038/s41375-018-0111-3)
Supplement: Supplementary file 1 — supplemental figures [file 41375_2018_111_MOESM1_ESM.pdf]

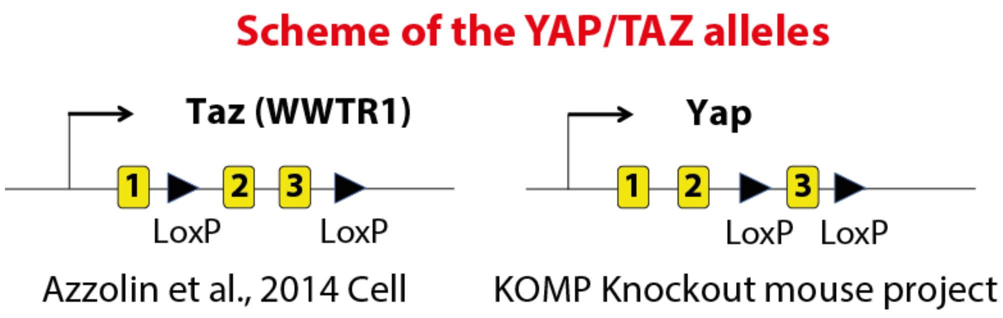

Supplementary Figure 1 Schematic view of the conditional alleles used in this study.

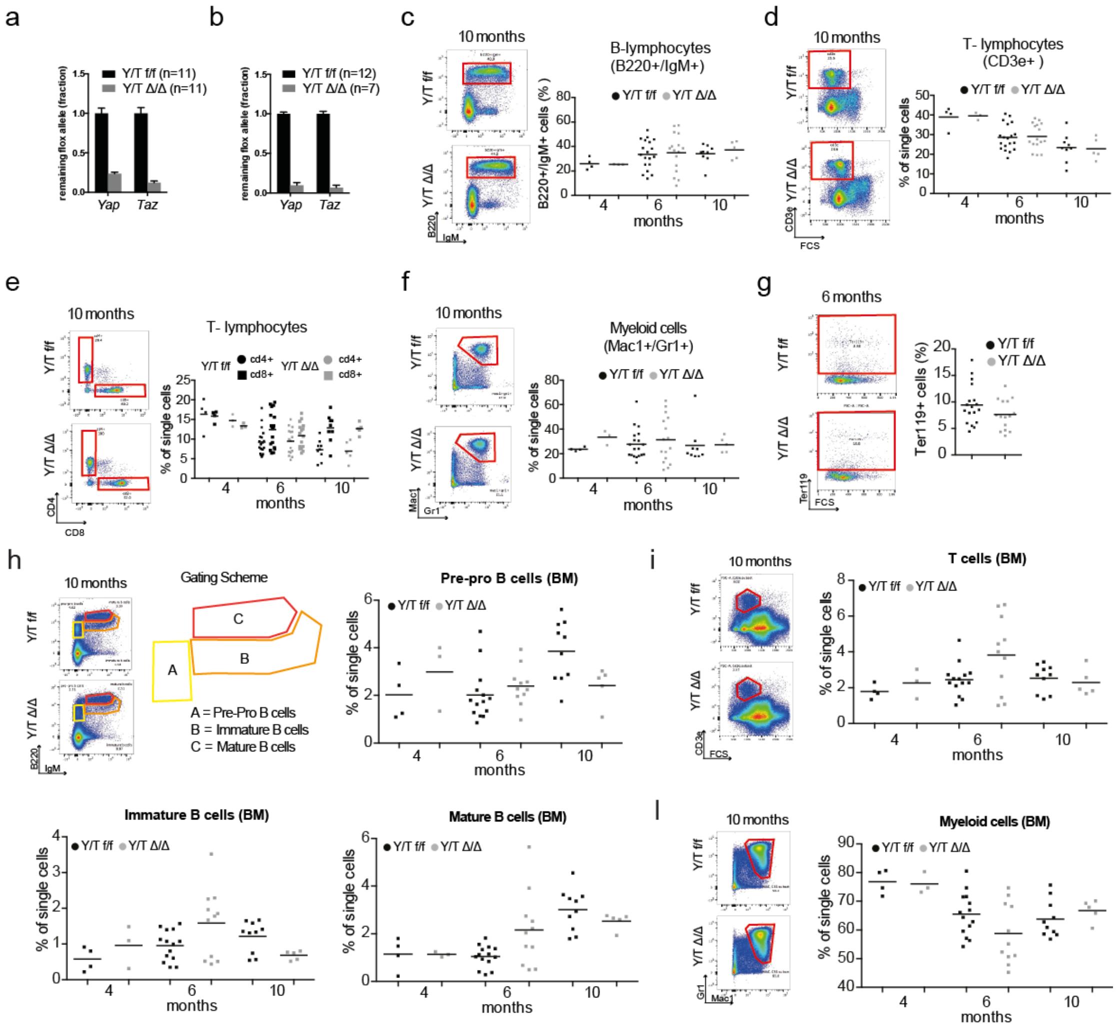

Supplementary Figure 2 Analysis of peripheral adult haematopoiesis upon Yap/Taz loss.

(a) qPCR analysis of CRE mediated recombination of the Yap and Taz alleles, determined on peripheral blood one month post *Mx1-CRE* activation.

(b) qPCR analysis of CRE mediated recombination of the Yap and Taz alleles, determined in bone marrow cells 10 months post *Mx1-CRE* activation.

(c-f) FACS analysis on peripheral blood from *Yap*$^{flox/flox}$/*Taz*$^{flox/flox}$ (Y/T f/f) (black) or *Yap*$^{\Delta/\Delta}$/*Taz*$^{\Delta/\Delta}$(Y/T Δ/Δ) (grey) mice at different time points after Mx1-CRE mediated recombination (4-6-10 months). FACS analysis of (c) B-cell lineage (B220+ IgM+), (d,e) T-cell lineage (CD3e+CD4+, CD3e+CD8a+),

(f) myeloid lineage (Mac1+ Gr1+). (g) FACS analysis on erythroid progenitors (Ter119+) 6 months after CRE mediated recombination.

(h-l) FACS analysis of total bone marrow from $Yap^{flox/flox}/Taz^{flox/flox}$ (Y/T f/f) (black) or $Yap^{\Delta/\Delta}/Taz^{\Delta/\Delta}$(Y/T Δ/Δ) (grey) mice at different time points after Mx1-CRE mediated recombination (4-6-10 months). FACS analysis (h) of B-cell lineage: pre-pro B cells (B220med IgM-), immature B cells (B220med IgM+), mature B cells (B220high IgM+), (i) T cell lineage (CD3e+), (l) myeloid cells (Mac1+ Gr1+). *P value less than 0.05. FACS Gating scheme is shown on the left side of ea

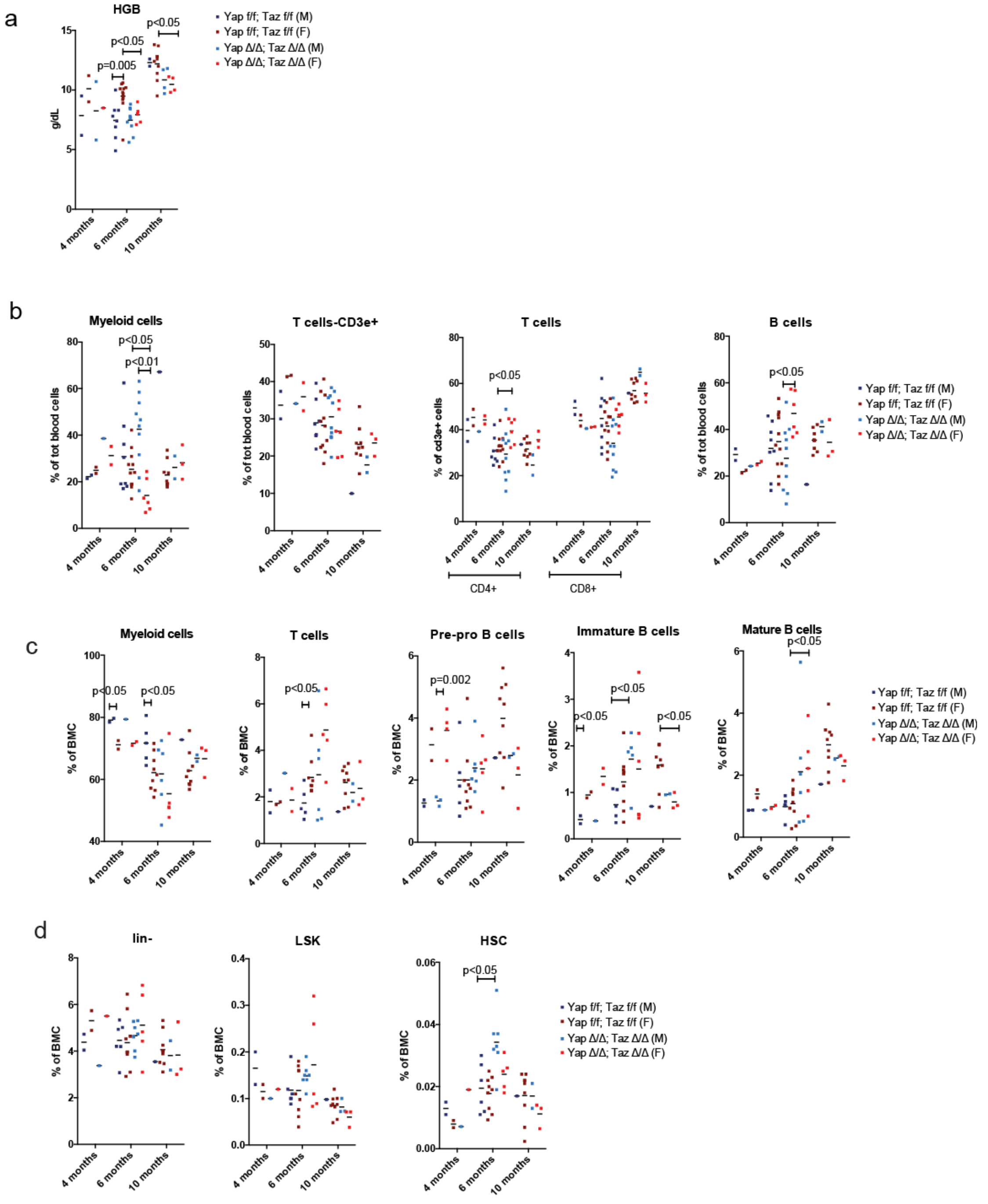

Supplementary Figure 3 Yap/Taz loss has no sex-specific effect on murine adult haematopoiesis.

(a)Peripheral blood analysis (b) and FACS analysis on peripheral blood and (c,d) total bone marrow at 4, 6 and 10 months post Mx1-CRE mediated

recombination in $Yap^{flox/flox}/Taz^{flox/flox}$ (Y/T f/f) (dark) or $Yap^{\Delta/\Delta}/Taz^{\Delta/\Delta}$(Y/T Δ/Δ) (light) male (blue) and female (red) mice.

(a) Quantification of Hemoglobin (HGB) in peripheral blood

(b) FACS analyses of myeloid cells (Mac1+Gr1+), T-cells (CD3e+, CD3e+CD4+, CD3e+CD8+) and B-cells (B220+IgM+) isolated from peripheral blood. (c,d) FACS analyses of bone marrow derived cells (c) myeloid cells (Mac1+Gr1+), T-cells (CD3e) and B-cells (pre-pro B cells: B220med IgM-, immature B cells: B220med IgM+, mature B cells: B220high IgM+). (d) FACS analyses of progenitors (Lin-: Lineage negative; LSK: Lin-Sca1+Kit+) and stem cells (HSCs: Lin- S+K+CD150+CD48-CD34-).

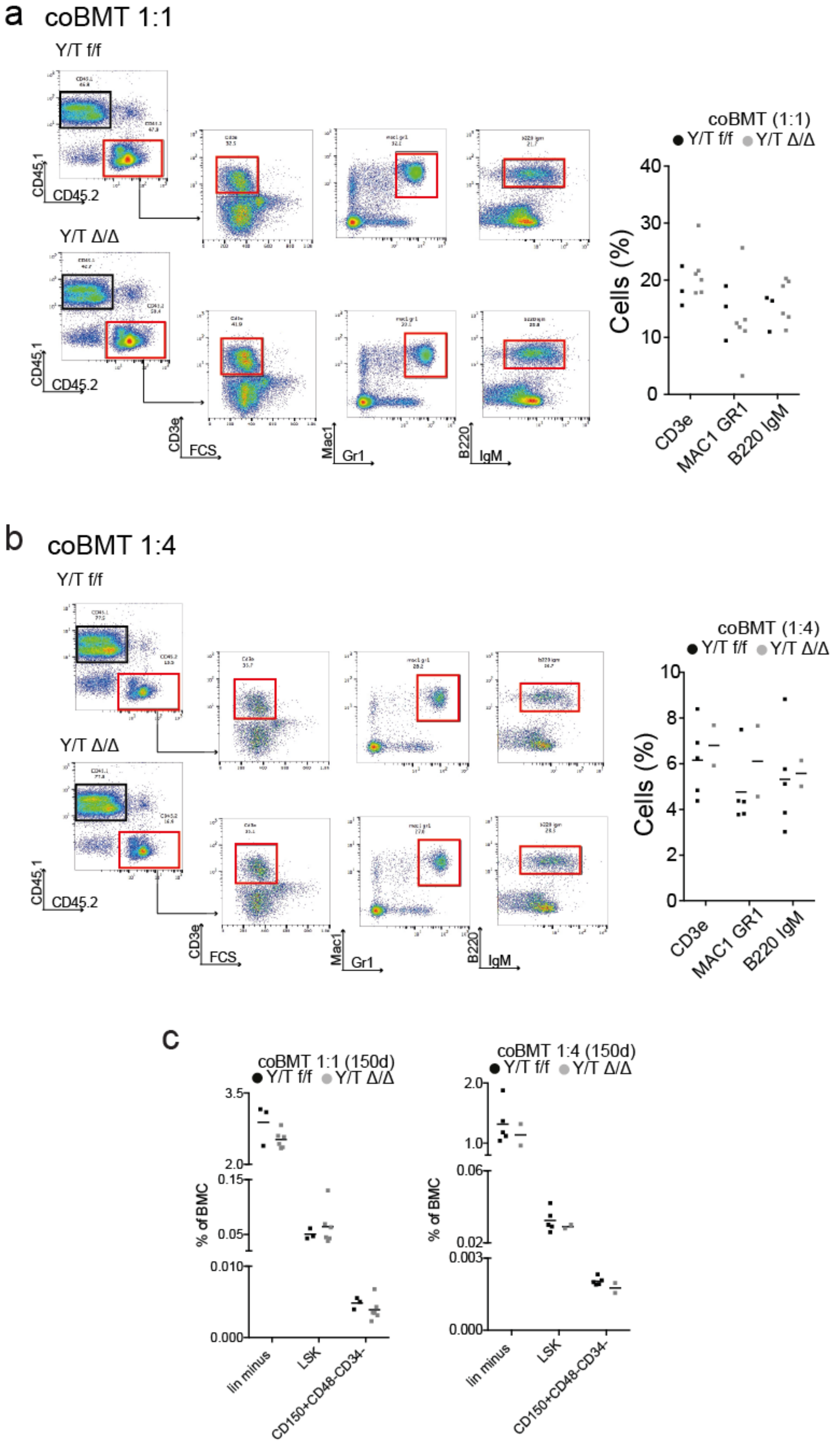
a
coBMT 1:1
Y/T f/f
Y/T Δ/Δ
CD45.1
CD45.2
CD3e
FCS
Mac1
Gr1
B220
IgM
coBMT (1:1)
Y/T f/f
Y/T Δ/Δ
Cells (%)
CD3e
MAC1 GR1
B220 IgM
b
coBMT 1:4
Y/T f/f
Y/T Δ/Δ
CD45.1
CD45.2
CD3e
FCS
Mac1
Gr1
B220
IgM
coBMT (1:4)
Y/T f/f
Y/T Δ/Δ
Cells (%)
CD3e
MAC1 GR1
B220 IgM
c
coBMT 1:1 (150d)
Y/T f/f
Y/T Δ/Δ
% of BMC
lin minus
LSK
CD150+CD48-CD34-
coBMT 1:4 (150d)
Y/T f/f
Y/T Δ/Δ
% of BMC
lin minus
LSK
CD150+CD48-CD34-

Supplementary Figure 4 Loss of *Yap/Taz* does not affect bone marrow transplantation.

Competitive bone marrow transplant (coBMT) experiment: BMCs from *Yap*$^{flox/flox}$/*Taz*$^{flox/flox}$ (Y/T f/f) or *Yap*$^{\Delta/\Delta}$/*Taz*$^{\Delta/\Delta}$(Y/T Δ/Δ) mice (CD45.2 positive) were mixed 1:1 or 1:4 with wild-type cells (CD45.1 positive). Peripheral haematopoiesis (T-cell lineage: CD3e+, myeloid lineage: Mac1+Gr1+, B-cell lineage: B220+IgM+) assessed by FACS on CD45.2 positive cells. Data are reported as percentage of total cells of each lineage (CD45.1 and CD45.2). (a) 1:1 mix. (b) 1:4 mix. FACS Gating scheme is shown on the left side of each panel.

(c) Analyses on progenitors and stem cells assessed by FACS on bone marrow derived CD45.2 positive cells. Data are reported as percentage of total cells of each lineage (CD45.1 and CD45.2). Lin-: Lineage negative; LSK: Lin-Sca1+Kit+; HSCs: Lin- Sca1+Kit+CD150+CD48-CD34-.

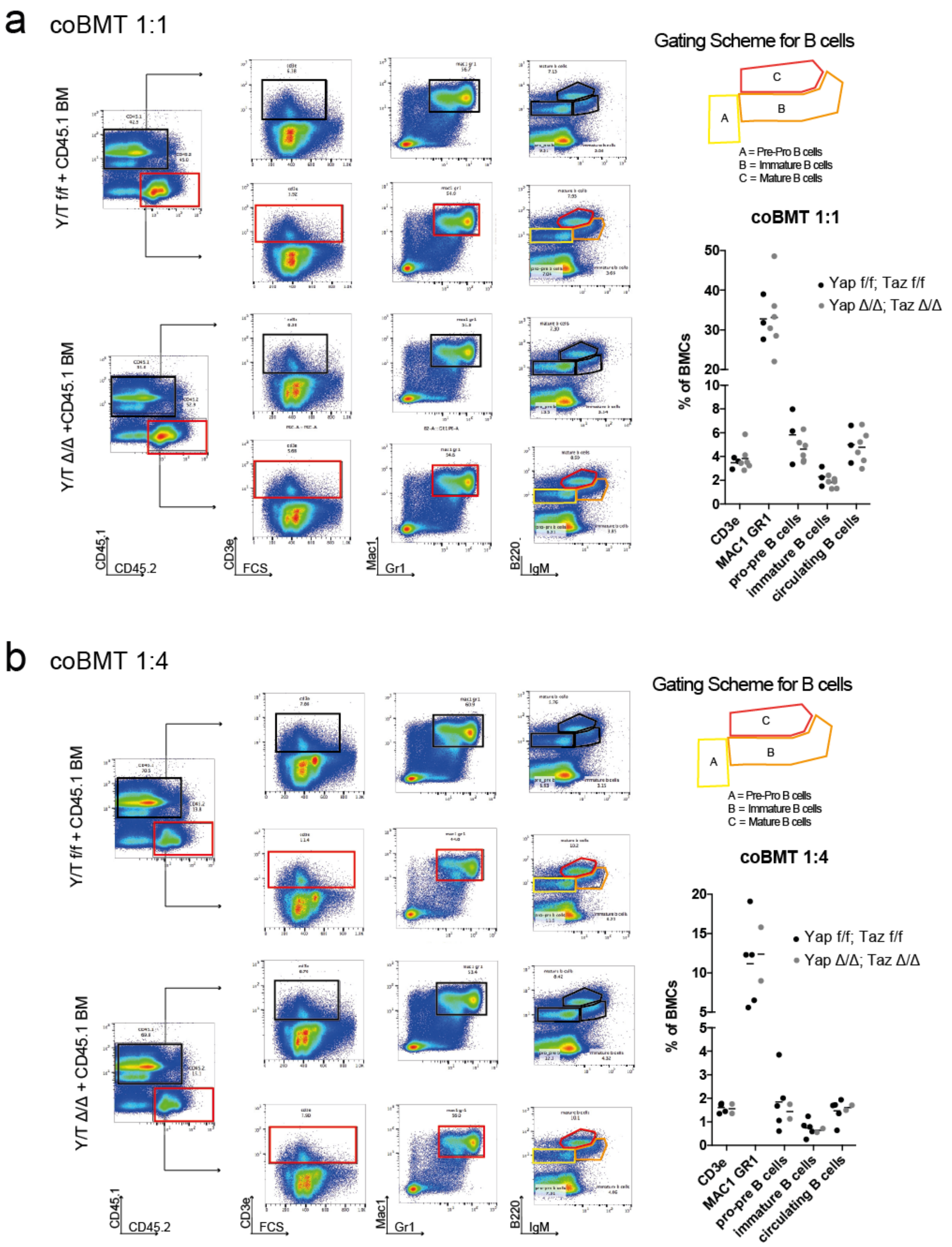

Supplementary Figure 5 CoBMT: chimerism analysis of bone marrow cells. (a,b) Bone marrow haematopoiesis assessed by FACS on CD45.2 positive cells after bone marrow transplantation into CD45.1 mice of *Yap*$^{flox/flox}$/*Taz*$^{flox/flox}$ (Y/T f/f) (black) or *Yap*$^{\Delta/\Delta}$/*Taz*$^{\Delta/\Delta}$(Y/T Δ/Δ) (grey) bone

marrow (CD45.2 positive) mixed 1:1 (a) or 1:4 (b) with wild-type cells (CD45.1 positive). Data are reported as % of total cells (CD45.1 and CD45.2) determined for each lineage: T cell lineage (CD3e+); Myeloid lineage (Mac1+Gr1+); B cell lineage (pre-pro B cells: B220med IgM-, immature B cells: B220med IgM+, mature B cells: B220high IgM+)

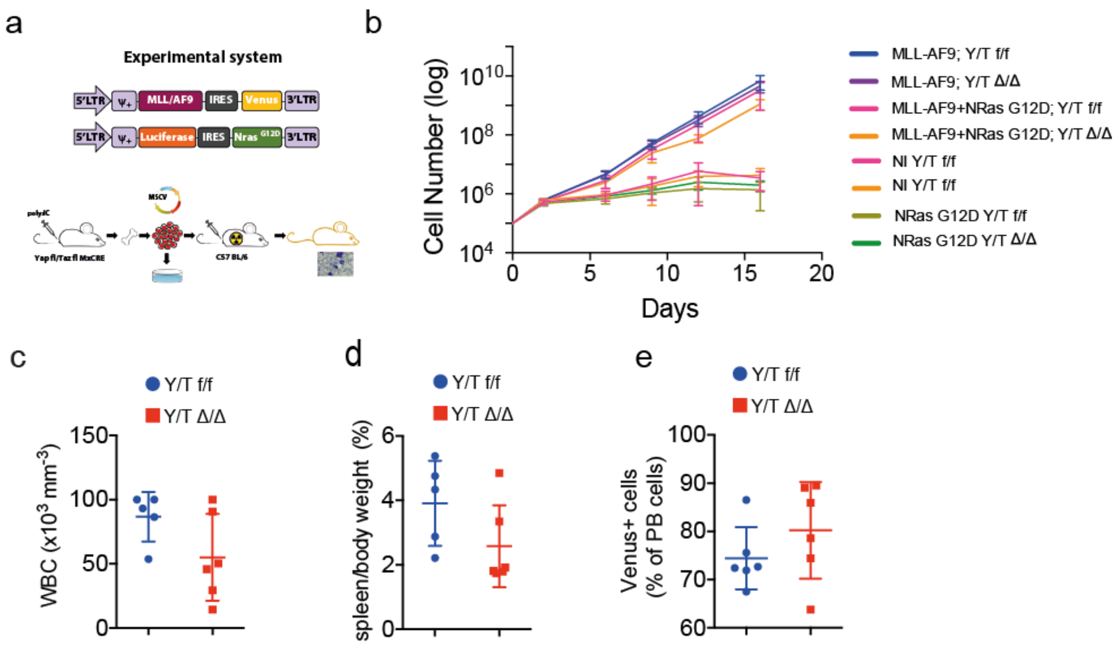

Supplementary Figure 6 Yap/Taz do not contribute to MLL-AF9 driven leukemia.

(a) Scheme of the experimental AML model

(b) Growth curves of Lin- cells transduced with the indicated oncogenes

(c-e) analysis of AMLs developed in mice transplanted with MLL-AF9 HPCs.

(c) WBCs counts, average values and standard deviations are shown (n=6)

(d) Spleen weight, average values and standard deviations are shown (n=6)

(e) Evaluation of circulating leukemic cells (Venus positive).
