# Supplementary material for: YAP and TAZ are dispensable for physiological and malignant haematopoiesis
Source: Leukemia. 2018 Mar 26;32(9):2037–40. doi: 10.1038/s41375-018-0111-3 (PMC6127083; doi:10.1038/s41375-018-0111-3)
Supplement: Supplementary file 2 — supplemental material and methods [file 41375_2018_111_MOESM2_ESM.docx]

**Materials and methods**

**Mice**

*Yap^flox/flox^/Taz^flox/flox^* murine line (Supplementary Figure 1) was obtained crossing *Yap^flox/flox^* mice (KOMP Knockout mouse project) and *Taz^flox/flox^* mice ^23^, a kind gift from Dr. S. Piccolo. Genomic DNA extracted from tail biopsies was used to evaluate offspring genotype. The primers used are listed in Supplementary table 1. For in vivo deletion of YAP and TAZ, 8 weeks old Mx1-CRE; *Yap^flox/flox^/Taz^flox/flox^* and *Yap^flox/flox^/Taz^flox/flox^* mice were subjected to three Poly(I:C) intra-peritoneal injections (250µg/mouse, one injection every two days). CRE mediated recombination was evaluated by qPCR analysis of genomic DNA derived from peripheral blood 30 days after the first injection. Specific primers annealing the loxP-flanked segment were used in order to determine Yap and Taz gene copy number by qPCR. Values were calculated using the Δ^Δct^ method. Values were normalized to Gapdh.

**Analysis on peripheral blood and stem/progenitor compartment**

Peripheral blood was collected from the tail vein. Total blood was analyzed using blood analyzer (Beckmann Coulter Ac-T 5diff). For lineage analysis by FACS, red blood cells were lysed using a hypotonic buffer (NH_4_Cl 150 mM, KHCO_3_ 10 mM, EDTA 100 μM) for 5 minutes on ice. Cells were subsequently washed and resuspended in 1% BSA in PBS buffer and stained with specific FACS antibodies for 1 hour on ice (Supplementary table 1). Bone marrow cells (BMCs) derived from tibias, femurs and iliac wings, were processed similarly. 30 x 10^6^ live bone marrow cells were used for the analysis of stem and progenitor compartment, 10^7^ live cells were used for the lineage analysis. Samples were acquired with MACSquant (Miltenyi Biotech).

**Bone marrow transplantation**

Bone Marrow Mononuclear cells (BMCs) were isolated using Histopaque 1083 (Sigma-Aldrich) according to manufacture’s instructions. For competitive transplants, cells from CD45.1 competitor mice were mixed with BMCs isolated from either *Yap^flox/flox^/Taz^flox/flox^* or *Yap^Δ/Δ^/Taz^Δ/Δ^* CD45.2 experimental mice (2 males and 2 females): live cells were counted using Trypan Blue and CD45.2 and CD45.1 BMCs were mixed in order to obtain 1:1 (CD45.2:CD45.1) and 1:4 (CD45.2:CD45.1) ratio. The expected chimerism was tested before transplantation by FACS analysis. A total of 10^6^ BMCs were transplanted into lethally irradiated (9 Gy) 8 weeks old CD45.1 female mice (10 mice/group), via tail vein injection. Long-term reconstitution was monitored after 60 days from transplantation on peripheral blood. Blood chimerism was analyzed by FACS, starting from 60 days post bone marrow transplantation. Primary coBMT was performed twice, for a total of 20 mice/experimental group. Secondary coBMTs were performed using as donor cells, BMCs isolated from primary transplants (5 x 10^6^ BMCs/mouse).

***Lineage depletion, retroviral transduction and transplantation***

Retroviral constructs pMSCV-MLL-AF9 ires-Venus and pMSCV-Luci-ires-RAS^G12D^ were kindly provided by Dr. Johannes Zuber. Bone marrow cells were lineage depleted using a lineage depletion kit (Miltenyi Biotech, Gladbach, Germany) and MACS magnetic bead technology (Miltenyi Biotech). Lineage depleted (Lin-) cells were grown in RPMI supplemented with 10% FBS (STEMCELL Technologies, cat # 06200), 2% L-Glutamine, 100 U/ml penicillin/streptomycin; 50 ug/ml SCF; 10 ng/ml IL3; 10 ng/ml IL6 and were trasduced with three rounds of infection. Venus expression was assesses 24h after the last infection by FACS (MACS Quant Analyzer, Miltenyi Biotech). 8 x 10^5^ Lin- cells were transplanted by tail vein injection into 6-8 weeks old lethally irradiated C57BL/6 recipient mice (7.0 Gy, in a single dose administered 24h prior to transplantation). Each mouse received a pool of Lin- cells derived from two independent donors (3 donors were injected with the same Lin- pool).

***Characterization of murine AML***

C57BL/6 recipient mice were sacrificed at terminal disease stage. Statistical evaluation of tumor free survival was based on the log rank (Mantel-Cox) test for comparison of the Kaplan-Meier event time format. Leukemic cells harvested from ether bone marrow or spleen were filtered through a 100 μm cell strainer (BD Falcon) to obtain single cell suspensions. For FACS analysis, harvested cells were first subjected to hypotonic shock and then were labeled for 20 minutes with a mix of lineage specific antibodies (B220, Mac-1, Gr-1, CD3e conjugated with PeCy7), with anti Sca-1 and anti c-kit antibodies diluted in MACS buffer (PBS, BSA 0,5%, EDTA 2mM).

***Cell proliferation and Colony-forming Unit (CFU) assay***

Cell proliferation assays of Lin- cells transduced with different combination of oncogenes were carried out by plating 100,000 cells per well, in 24-well format, in triplicates. Cell growth was assessed by FACS cell counting, every 2 days for up to 16 days (MACS Quant Analyzer, Miltenyi Biotech).

For CFU assay, cells were cultured in MethoCult^TM^ (GF M3236, STEMCELL Technologies). Briefly, 2 x 10^3^ cells were seeded in triplicates in 24 well plates containing 1mL of methylcellulose culture media and incubated at 37 °C with 5% CO2. The progenitor cells formed individual colonies that were counted with an inverted microscope on day 14 of culture.

***Statistical analyses***

FACS data were analyzed with FlowJo (Version 9.1). Multiple T-test was used to evaluate statistical significance, comparisons were marked only if statistically significant (*: P < 0.05). Analysis and graphs were generated using Prism (GraphPad Software). Proliferation and CFU assays are presented as means ± standard error of mean (SEM) of triplicates from two independent experiments.
